# Intrinsic capacity across the adult lifespan in China: baseline analysis from the nationwide longitudinal PENG ZU cohort

**DOI:** 10.1007/s11357-026-02111-3

**Published:** 2026-02-25

**Authors:** Ju Cui, Yujia Gu, Jing Pang, Huafang Gao, Li-Qun Zhang, Xiaolan Wu, Junwu Dang, Juan Li, Xinyi Zhu, Senlin Luo, Zhao Wang, Fuyi Tu, Likun Zhang, Yang Liu, Guangyu Yang, Qin Zhang, Wanxia Wang, Wen Tian, Weimin Li, Wei Xiong, Xuewei Zhang, Zhanyi Lin, Songbai Zheng, Lunzhi Dai, Xiaoming Wang, Zhongqiu Lu, Xiaohong Sun, Jianping Cai, Wei Ma, Tiemei Zhang

**Affiliations:** 1 The Key Laboratory of Geriatrics, Beijing Institute of Geriatrics, Institute of Geriatric Medicine, Chinese Academy of Medical Science, Beijing Hospital/National Center of Gerontology of National Health Commission, Beijing, P.R. China; 2 Institute of Statistics and Big Data, Renmin University of China, Beijing, P.R. China; 3 National Human Genetic Resources Center, National Research Institute for Family Planning, Beijing, P.R. China; 4 Institute of Healthy Ageing, China Research Center on Ageing, Beijing, P.R. China; 5 Center on Aging Psychology, CAS Key Laboratory of Mental Health, Institute of Psychology, Chinese Academy of Sciences, Beijing, P.R. China; 6 Department of Psychology, University of Chinese Academy of Sciences, Beijing, China; 7 Beijing Institute of Technology, Beijing, P.R. China; 8 Department of Pharmacology, School of Pharmaceutical Sciences, Tsinghua University, Beijing, P.R. China; 9 Department of Geriatrics, The First Affiliated Hospital, School of Medicine, Zhejiang University, Hangzhou, P.R. China; 10 The Institute of Clinical Research and Translational Medicine, NHC Key Laboratory of Diagnosis and Therapy of Gastrointestinal Tumor, Gansu Provincial Hospital, Lanzhou, P.R. China; 11 Department of Geriatrics, The First Affiliated Hospital of China Medical University, Liaoning Provincial Clinical Research Center of Geriatric Disease, Shenyang, P.R. China; 12 Beijing Tuberculosis and Thoracic Tumor Research Institute, Beijing Chest Hospital, Capital Medical University, Beijing, China; 13 Department of Geriatrics, Southwest Hospital, The First Hospital Affiliated to Army Medical University, Chongqing, P.R. China; 14 Health Management Center, Xiangya Hospital, Central South University, Changsha, China; 15 Department of Geriatrics, Guangdong Provincial Geriatrics Institute, Guangdong Provincial People’s Hospital, Guangdong Academy of Medical Sciences, Southern Medical University, Guangzhou, P.R. China; 16 Geriatric Medicine Department, Huadong Hospital Affiliated to Fudan University, Shanghai, P.R. China; 17 National Clinical Research Center for Geriatrics, State Key Laboratory of Biotherapy, West China Hospital, Sichuan University, Chengdu, 610041 China; 18 Department of Geriatrics, Xijing Hospital, Air Force Medical University, Xi’an, 710032 China; 19 Department of Emergency Medicine, The First Affiliated Hospital of Wenzhou Medical University, Wenzhou, China; 20 Wenzhou Key Laboratory of Emergency and Disaster Medicine, Wenzhou, 325000 China; 21 Department of Geriatrics, Peking Union Medical College, Chinese Academy of Medical Sciences, Peking Union Medical College Hospital, No. 1 Shuaifuyuan, Dong Cheng District, Beijing, 100730 P.R. China

**Keywords:** Intrinsic capacity, Aging, Age-related decline, Health consciousness, PENG ZU study, China

## Abstract

Intrinsic capacity (IC), a composite measure of physical and mental capacities, reflects the functional manifestations of biological aging, yet large-scale population evidence on how IC varies throughout adulthood outside Western settings remains limited. Using data from 17,086 Chinese adults aged 25–89 years in the nationally representative PENG ZU cohort, we characterized age-related variation in IC and its associated factors. IC was assessed across five domains (locomotion, cognition, vitality, psychological, sensory) using self-reported data and physical performance tests. A composite IC score was generated using weighted domain scores and standardized to a 0–100 range. IC levels declined progressively with age, with a sharper decrease and greater variability observed after age 60 in both sexes. The mean IC score was 67.8 (SD = 14) for participants aged < 60 years and 48.6 (SD = 17) for those aged ≥ 60 years. Inflection points were identified at age 60 for men and 62 for women, with significantly faster decline thereafter. The coefficient of variation in IC scores increased with age and differed significantly between adjacent age groups after 60 years. Among those aged ≥ 60, only 6.0% retained high IC, whereas nearly one-third experienced moderate-to-severe loss. Locomotion, cognition, vitality, and sensory domains showed marked age-related decline, with the largest reductions in cognition (60.7%), followed by locomotion (52.9%), vitality (51.0%), and sensory function (39.5%) between ages 50–54 and 85–89, while the psychological domain remained stable. Multivariate ANOVA identified age, education, and health consciousness (assessed via self-reported beliefs about personal responsibility for maintaining health) as the top factors associated with IC. Older age was associated with lower IC, whereas higher education and greater health consciousness were associated with higher IC. Furthermore, health consciousness was inversely associated with ADL/IADL disability across all age groups. These findings identify around 60 years as a critical window for the preservation of intrinsic capacity in Chinese adults and highlight health consciousness as a promising modifiable correlate that merits further investigation in the context of healthy aging.

**Graphical Abstract:**

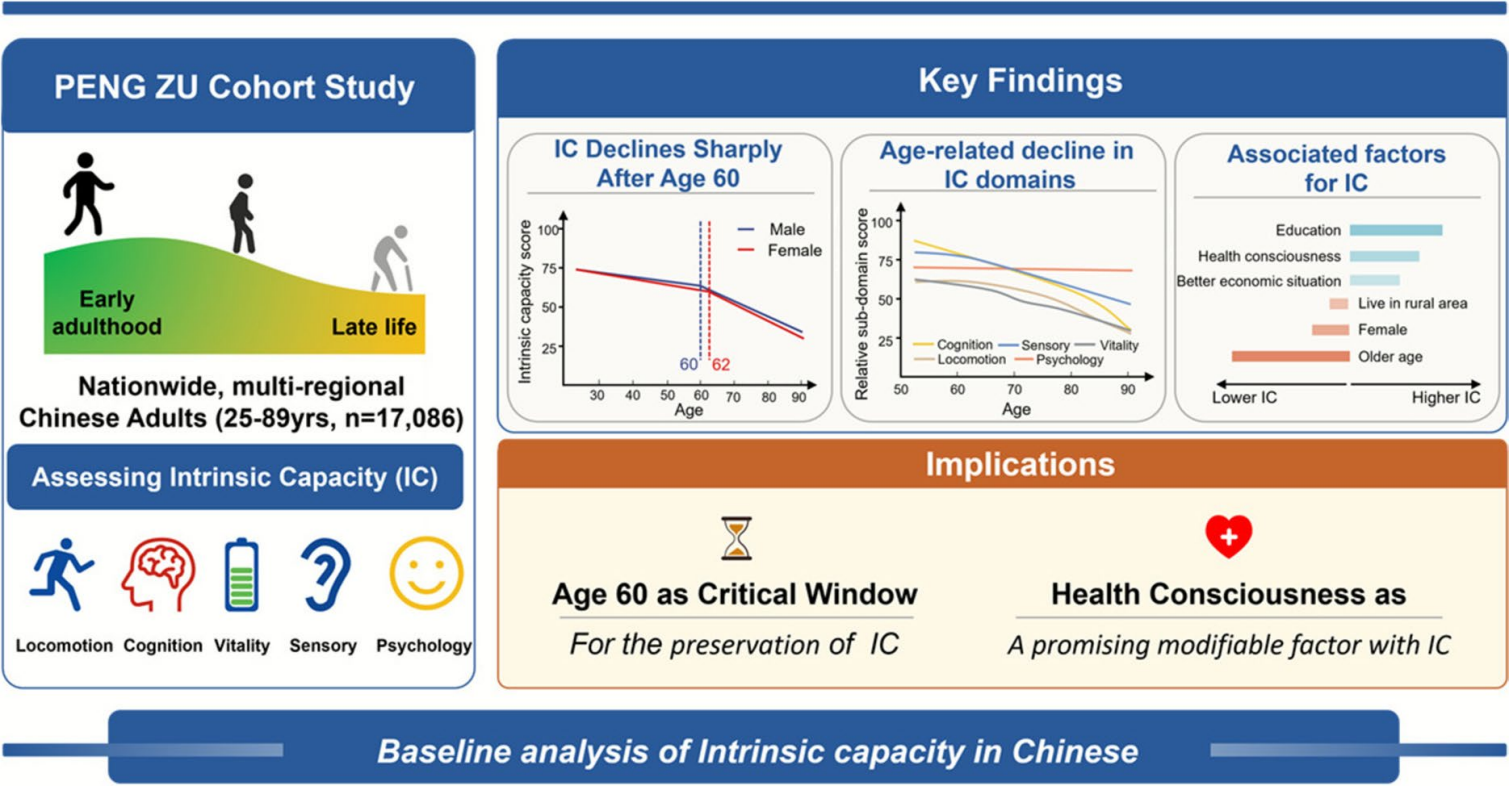

**Supplementary Information:**

The online version contains supplementary material available at 10.1007/s11357-026-02111-3.

## Introduction

The United Nations declared 2021–2030 the Decade of Healthy Ageing [1], highlighting the urgent need to improve health systems to meet the changes of an aging population. Achieving this goal requires not only a clear understanding of the concept of healthy aging but also foundational data on health status and effective tools for health assessment and intervention.

To address this need, the World Health Organization (WHO) introduced the concept of Intrinsic Capacity (IC), defined as the composite of all the physical and mental capacities that an individual can draw upon, reflecting their underlying potential to function [2]. The concept of IC provides a new dimension for understanding health in terms of body functions and shifts the concept of human health from a disease-centered approach to a comprehensive analysis of functional status [3]. This paradigm shift is essential for addressing the health demands of the aging population, which is increasing significantly throughout the world [4].

The maintenance of IC is one of the core concepts of healthy aging proposed by the WHO, aiming to strengthen and maintain the functional ability in later life [2]. More recently, IC has been considered a determinant of physical resilience and functions as an important target for interventions aimed at enhancing older adults’ ability to withstand stressors and maintain independence [5, 6]. Therefore, the assessment of IC and the development of effective interventions to slow or even reverse the decline of IC will be of great significance for promoting healthy aging.

According to the WHO guidelines, IC is typically assessed across five domains: locomotor, sensory, vitality, cognition, and psychology [3]. Many studies have explored the measurement of IC and its association with various health outcomes. Recent reviews by Beyene et al. [7] and Sanchez-Rodriguez et al. [8] summarized available assessment tools. However, there is currently no universally accepted standard for selecting appropriate indicators within each domain or integrating domain scores to derive a composite IC score. While most studies adopt the WHO’s five domain model, alternative frameworks have been proposed, such as dividing the sensory domain into vision and hearing (six domains) [9], adding continence (seven domains) [10], incorporating metabolic, inflammatory, or lipid-related indicators (eight domains) [11] and lacing the sensory [12]/cognition domain [13] (four domains). Some even ignore domains and sum all indicators. Correspondingly, IC calculation methods vary, ranging from empirical summation and standardized averaging to more complex data-driven approaches like factor analysis [14, 15], principal component analysis [16] and latent variable modeling [9]. This diversity underscores the need for a clinically practical and interpretable IC metric.

Most existing IC studies have focused on individuals aged 60 years and older, consistently demonstrating associations between low IC and adverse outcomes such as comorbidity [17], frailty [18], disability [6], and mortality [19, 20]. Older age, women, low education, low income, unmarried status, and urban living are linked to lower intrinsic capacity [14, 16, 21–23]. Lifestyle interventions such as exercise and nutrition improved IC [24]. All these factors are objective characteristics, and subjective factors have been largely overlooked. Furthermore, evidence on how IC varies across the full span of adulthood remains limited. One recent study by Lu et al., based on the INSPIRE-T cohort in France, established reference centiles for IC across adults aged 20–102 years, revealing a marked decline in IC scores after age 65 [25]. Nevertheless, domain-specific age-related patterns remain unclear, and similar life course approaches are lacking in Asian populations, particularly in middle-income countries.

In addition, the methods of IC scoring may affect its interpretation. The commonly used sum/mean score approach, which equally weights each domain, may obscure the fact that different domains contribute unequally to overall IC. Conversely, the factor score method models domains as orthogonal constructs, enhancing statistical rigor but often at the expense of clinical interpretability.

To address these gaps, the present study applied the entropy weight method (EWM) to derive a data-driven and interpretable composite IC score based on baseline data from the PENG ZU cohort, a large community-based sample of adults aged 25–89 years from seven major geographical areas in China. This study aimed to (1) examine the age-related variation patterns of the IC and its constituent domains across adulthood in the Chinese population; (2) explore the relative contributions of each domain to overall IC; and (3) identify modifiable factors associated with IC levels. These findings are expected to enhance our understanding of the age-related patterns of IC across the life course, provide evidence for early identification of at-risk individuals, and inform the design of targeted interventions to promote healthy aging.

## Subjects and methods

### Data source and study design

This study used data from the PENG ZU Study on Healthy Ageing in China (PENG ZU Cohort, an ongoing study from 2018 to date). The objectives and methodology of the PENG ZU cohort have been published previously [26]. In summary, the PENG ZU cohort aims to examine changes in multidimensional health status throughout adulthood and to clarify the dynamic processes associated with aging. The PENG ZU cohort protocol has been approved by the Research Ethics Committee of Beijing Hospital (2019BJYYEC-054-02), and informed consent was obtained from all participants. This study primarily conducted a cross-sectional analysis based on baseline data from 17,086 participants in the PENG ZU Study, all of whom had complete information on intrinsic capacity, functional ability, and clinical indicators. In addition, a subset of 5019 participants aged 60 years and older, whose baseline assessments were conducted in 2019–2020, were followed up in 2022 for an average of 26 months. Mortality information was obtained through active follow-up.

### Measurement of intrinsic capacity

The selection criteria for the assessed indicators were as follows: (a) the indicators showed strong correlations with other performance-based or self-reported measures within the corresponding domain of our dataset, as confirmed by correlation analysis; (b) the indicators were feasible and easy to administer. Balancing the consistency with previous IC studies and the convenience of large-scale population assessment, we selected the following variables to operationalize the IC score.

*Locomotion*: Locomotion refers to the active movement of the body (with or without assistive devices), which is an essential ability for an individual to participate in various activities. The participants were asked to walk at their usual walking pace, start from the same position, and the test ends when the first foot crosses the 6-m line. According to the percentile distribution, gait speed < 0.6 m/s, it is scored as 1 point; 0.6 m/s ≤ gait speed < 0.9 m/s, it is scored as 2 points; 0.9 m/s ≤ gait speed < 1.3 m/s, it is scored as 3 points; gait speed ≥ 1.3 m/s, it is scored as 4 points.

*Cognition*: The cognitive ability was measured using the Montreal Cognitive Assessment 5-minute protocol (MoCA 5-min protocol) [27]. It includes 4 items examining attention, executive ability/verbal skills, orientation, and memory, with a total score of 30 points. A total score of ≥ 24 indicates normal cognitive function and is assigned 3 points; a score between 15 and 23 indicates impaired cognitive function and is assigned 2 points; and a score of < 15 indicates low cognitive function and is assigned 1 point. This test was conducted only among adults aged 50 years and above, and for participants under 50 years, cognitive capacity was imputed as the highest score.

*Vitality*: Vitality was assessed with BMI and hand grip strength (Kg). BMI was calculated as weight (kg) divided by the square of height (m^2^). Normal weight (BMI between 18.5 and 24.9) was assigned 4 points, first-degree overweight 3 points (a BMI between 24.9 and 26.9), second-degree overweight 2 points (a BMI between 26.9 and 29.9), and obesity (a BMI greater than 29.9) was assigned 1 point. The maximum dominant hand grip strength was measured. Due to the obvious gender differences in muscle mass and strength, grip strength scores for men and women are evaluated by using different standards, confirmed by gender-specific quartile-based cutoffs: for men, grip strength < 20 kg (kg) scores 1 point, 20 kg ≤ grip strength < 30 kg scores 2 points, 30 kg ≤ grip strength < 40 kg scores 3 points, grip strength ≥ 40 kg scores 4 points; for women, grip strength < 15 kg scores 1 point, 15 kg ≤ grip strength < 22 kg scores 2 points, 22 kg ≤ grip strength < 30 kg scores 3 points, grip strength ≥ 30 kg scores 4 points.

*Psychology*: The psychological state is evaluated from three aspects: self-assessment of emotional state, self-rated life satisfaction, and self-rated health satisfaction. The self-assessment of emotional state was measured using the DASS-21, which evaluates the psychological states of depression, anxiety, and stress [28]. Self-rated life and health satisfaction were measured using single-item 10-point self-report scales, with scores ranging from 1 (very unsatisfied) to 10 (very satisfied), where 9–10 scores 3 points, 6–8 scores 2 points, 3–5 scores 1 point, and 1–2 scores 0 points. DASS scales = D + A + S; D = 0: depression score > 9, D = 1: depression score ≤ 9; A = 0: anxiety score > 7, A = 1: anxiety score ≤ 7; S = 0: stress score > 14, S = 1: stress score ≤ 14; Psychological state score = DASS scales + 2·5 * self-rated life satisfaction + 20 * self-rated health satisfaction.

*Sensory*: Self-reported hearing status was recorded. Question: “Have you experienced any hearing loss in the past one year?” Three predefined response options: (1) Answer Options: (1) Hearing with no decline (3 points); (2) hearing with some decline but not affecting daily life (2 points); (3) hearing with decline affecting daily life (1 point).

The detailed information for recoding the indicators is summarized in Table S1.

Conceptually inspired by the framework proposed by WHO [29], composite IC score was measured using the following four steps: (1) Data for all the domains were scaled down to values in the range of 0–1; (2) Entropy Weight Method (EWM) ^[30,31]^ was used to determine weights for five domains; (3) The original IC score was derived by the weighted sum of five domains; (4) After deriving the original IC score, the Box-Cox and linear transformations were applied to calculate the composite IC score in the range of 0–100, with higher scores indicating higher IC. Detailed information on the development of the composite intrinsic capacity score is presented in the Methods section of the Appendix and illustrated in Figure S1.

Domain score was defined as the value scaled to a 0–1 range in step (1) during the derivation of the IC composite score. Two types of scores were generated from the domain scores. The relative domain scores were calculated by dividing the specific domain value by the maximum value in that domain and then multiplying the resulting value by 100. To assess the intrinsic capacity of an individual using the radar graph, linear regression and generalized additive models were used to predict the continuous domain-specific scores.

Participants were categorized into four groups based on IC score cut-off ranges: high, sufficient, moderate loss, and significant loss. The cut-off ranges were determined using the mean and standard deviation of the IC scores, with the boundaries between adjacent levels adjusted for practical application. The details of classification were as follows: (1) high level: IC score ≥ 75 (approximately ≥ 1.5 SD above the mean); (2) sufficient level: 40 ≤ IC score < 75 (approximately between − 0.75 SD and 1.5 SD of the mean); (3) moderate loss: 20 ≤ IC score < 40 (approximately between − 2 SD and − 0.75 SD of the mean); (4) significant loss: IC score < 20 (approximately <  − 2 SD of the mean).

### Sociodemographic and modifiable factors

Sociodemographic factors, including sex, age, education, marital status, residence in urban or rural areas, were registered. Modifiable risk factors were evaluated using self-reported information about economic situation, convenient medical service, living alone, and health consciousness.

Economic situation was assessed through self-report using a five-point Likert scale: 1 = very wealthy, 2 = relatively wealthy, 3 = basically adequate, 4 = relatively difficult, and 5 = very difficult. For analytical purposes, responses of 1 and 2 were categorized as “low financial difficulty,” 3 as “moderate financial difficulty,” and 4 or 5 as “high financial difficulty.”

Convenient medical service was assessed by asking participants, “Do you think it is convenient for you to visit a hospital or clinic?” Responses were rated on a five-point scale: 1 = very convenient, 2 = relatively convenient, 3 = neutral, 4 = somewhat inconvenient, and 5 = very inconvenient. For analysis, responses of 1 and 2 were classified as convenient, 3 as neutral, and 4 and 5 as inconvenient.

Health consciousness was assessed by a questionnaire. Participants were asked to indicate which of the following statements best reflected their understanding of health: (1) health is defined as the absence of disease, injury, and disability; (2) health is primarily maintained through reliance on hospitals and physicians; (3) maintaining health is a personal responsibility; (4) health is best preserved through wellness and preventive practices. Response option (3) indicates good health consciousness, whereas all other response options reflect a lack of health consciousness.

### Health and functional measures

Self-reported chronic diseases, including hypertension, diabetes, heart disease, lung disease, chronic nephritis, cancer, stroke and other cerebrovascular diseases, dementia, Parkinson’s disease, arthritis, cataract, cervical and lumbar spine disorders, and metabolic disorders (elevated blood glucose, lipid, or uric acid levels), were recorded by asking participants if a physician had ever told them that they had such a condition.

The functional ability was evaluated through validated questionnaires assessing activities of daily living (ADL) and instrumental activities of daily living (IADL) [32, 33]. ADL assesses an individual’s ability to perform six basic self-care tasks, including transferring, eating, dressing, toileting, bathing, and decorating. For each activity in ADL, participants reported whether they could perform it by themselves (including self-care, mild dependence, moderate dependence, and severe dependence). IADL included seven activities essential to living independently in the community, including shopping for groceries, housekeeping, using transportation, using the telephone, taking medicines, handing finances, and making electronic payments. For each activity in IADL, participants reported whether they could perform it by themselves (including independent, needs some assistance, and completely dependent). For ADL, each item was scored according to the level of dependence, and the total score ranged from 0 to 24. Based on the total score, participants were classified as self-care (0–3), mildly dependent (4–8), moderately dependent (9–18), or severely dependent (≥ 19). For IADL, participants were categorized as good (able to perform all tasks), acceptable (unable to perform one task), reduced capacity (unable to perform two tasks), or lack of capacity (unable to perform three or more tasks).

Follow-up was conducted to record all-cause mortality among participants aged 60 years or older whose baseline assessments had been conducted more than two years prior. Hemoglobin was measured using an automatic hematology analyzer (Sysmex XN-20 system, Japan). Fasting blood glucose and hypersensitive C-reactive protein in serum were assessed using the Hitachi Automatic Analyzer (LABOSPECT 008 AS, Japan). Blood pressure was measured using an electronic sphygmomanometer (Yuwell, 680AR).

### Statistical analysis

Continuous variables are presented as mean ± SD. Categorical variables were presented as numbers or percentages. Discrete items are presented as a ratio in groups. Spearman correlation analysis and regression analysis were used for item selection, validation of the IC score, and identification of factors significantly associated with the IC score. Piecewise linear regression models were used to estimate the inflection points of composite IC scores [34, 35]. Restricted cubic spline (RCS) regression was used to find the potential non-linear association between the relative domain score and age. RCS models provide flexibility for capturing non-linear associations, though they may increase model complexity and reduce interpretability. In this study, spline functions with five knots were placed at predefined ages (35, 45, 55, 65, and 75 years), following standard recommendations. The Spearman partial correlation coefficient was used to construct the correlation network with the impact parameters of interest. As the sociodemographic and modifiable factors of interest were categorical, we used a multivariate ANOVA model, which corresponds to a linear regression with dummy-coded predictors. Prior to the ANOVA analysis, we evaluated multicollinearity using the generalized variance inflation factor (GVIF) and the condition number of the dummy-coded design matrix. In addition, a group lasso analysis was conducted as a robustness check to examine potential shrinkage effects on the factors. The association between each factor and the IC scores was evaluated using the overall *F*-test *p*-value for that indicator, and the indicators were then ranked according to these *p*-values, without considering interaction effects. Standardized effect sizes were also calculated to provide a more robust ranking of the factors. In addition, we performed an ordered linear regression to assess monotonic linear trends between these factors and the IC score. The coefficient of variation (CV) was calculated as the ratio of standard deviation to mean. It was used to evaluate the dispersion of the composite IC score and the relative domain score among participants. *p*-values were estimated using the *t*-test or the *F*-test. All statistical analyses were conducted using R software (version 4.2.0). All tests were two-sided, and *p*-values less than 0.05 were considered statistically significant.

## Results

### Age-related patterns of intrinsic capacity

General characteristics of the study participants are summarized in Table 1. A total of 17,086 participants (46.3% men and 53.7% women) with a mean age of 52.8 years (SD = 17) were included.

**Table 1:** General and IC assessment-related information of the study participants

Variables	Age groups				
	Age 25–49	Age 50–59	Age 60–69	Age 70–79	Age 80–89
*n*	8033	2801	2755	2188	1309
Female	4375/8033 (54.5%)	1583/2801 (56.5%)	1432/2755 (52.0%)	1143/2188(52.2%)	647/1309 (49.4%)
Residential areas					
Rural	1791/8033 (22.3%)	823/2801 (29.4%)	1096/2755 (39.8%)	984/2188 (45.0%)	618/1309 (47.2%)
Urban	6242/8033 (77.7%)	1978/2801 (70.6%)	1659/2755 (60.2%)	1204/2188 (55.0%)	691/1309 (52.8%)
Education					
Illiteracy	32/8033 (0.4%)	42/2801 (1.5%)	355/2755 (12.9%)	429/2188 (19.6%)	322/1309 (24.6%)
Primary	321/8033 (4.0%)	305/2801 (10.9%)	537/2755 (19.5%)	648/2188 (29.6%)	388/1309 (29.6%)
Secondary	3197/8033 (39.8%)	1873/2801(66.9%)	1504/2755(54.6%)	862/2188 (39.4%)	467/1309 (35.7%)
High	4483/8033 (55.8%)	581/2801 (20.7%)	359/2755 (13.0%)	249/2188 (11.4%)	132/1309 (10.1%)
Hearing lossing^1^					
1	6573/8033 (81.8%)	1641/2801 (58.6%)	1277/2755(46.4%)	693/2188 (31.7%)	261/1309 (19.9%)
2	1383/8033 (17.2%)	1130/2801 (40.3%)	1385/2755 (50.3%)	1295/2188 (59.2%)	798/1309 (61.0%)
3	77/8033 (1.0%)	30/2801 (1.1%)	93/2755 (3.4%)	200/2188 (9.1%)	250/1309 (19.1%)
BMI (kg/m^2^)	23.8 [3.8]	24.7 [3.5]	24.4 [3.4]	24.2 [3.6]	23.8 [3.8]
Grip strength (kg)	30.2 [11.7]	26.8 [10.0]	25.3 [10.0]	21.9 [9.0]	17.9 [8.2]
Walking speed (m/s)	1.1 [0.4]	1.1 [0.4]	1.0 [0.3]	0.9 [0.3]	0.8 [0.3]
MoCA 5-min Score	NA	25.1 [4.2]	22.8 [5.3]	20.4 [6.1]	17.1 [6.5]
Quality of life^2^	7.7 [2.0]	7.7 [1.9]	7.8 [1.7]	7.8 [1.6]	7.5 [1.8]
Health condition^3^	7.3 [2.0]	7.1 [1.9]	7.2 [1.8]	7.1 [1.7]	7.0 [1.8]
DASS Score^4^					
Depression	6.9 [9.3]	3.5 [7.6]	2.7 [5.9]	3.0 [6.0]	2.6 [4.6]
Anxiety	7.0 [9.2]	3.7 [7.4]	3.2 [6.0]	3.6 [6.2]	3.3 [4.8]
Stress	8.3 [9.8]	4.3 [8.1]	3.5 [6.8]	3.4 [6.5]	2.8 [5.0]

Data are represented as *n*/N (%) or mean [SD]

^1^Hearing loss: 1 = no impairment

2 = have impairment but does not impact daily life

3 = have impairment and impacts on daily life

^2^Self-rating of the quality of life: score range: 0–10; score 10 = very satisfied; score 0 = very dissatisfied

^3^Self-rating of health: score range: 0–10; score 10 = very satisfied; score 0 = very dissatisfied

^4^Scores obtained from the Depression, Anxiety and Stress Scale

**Fig. 1:**
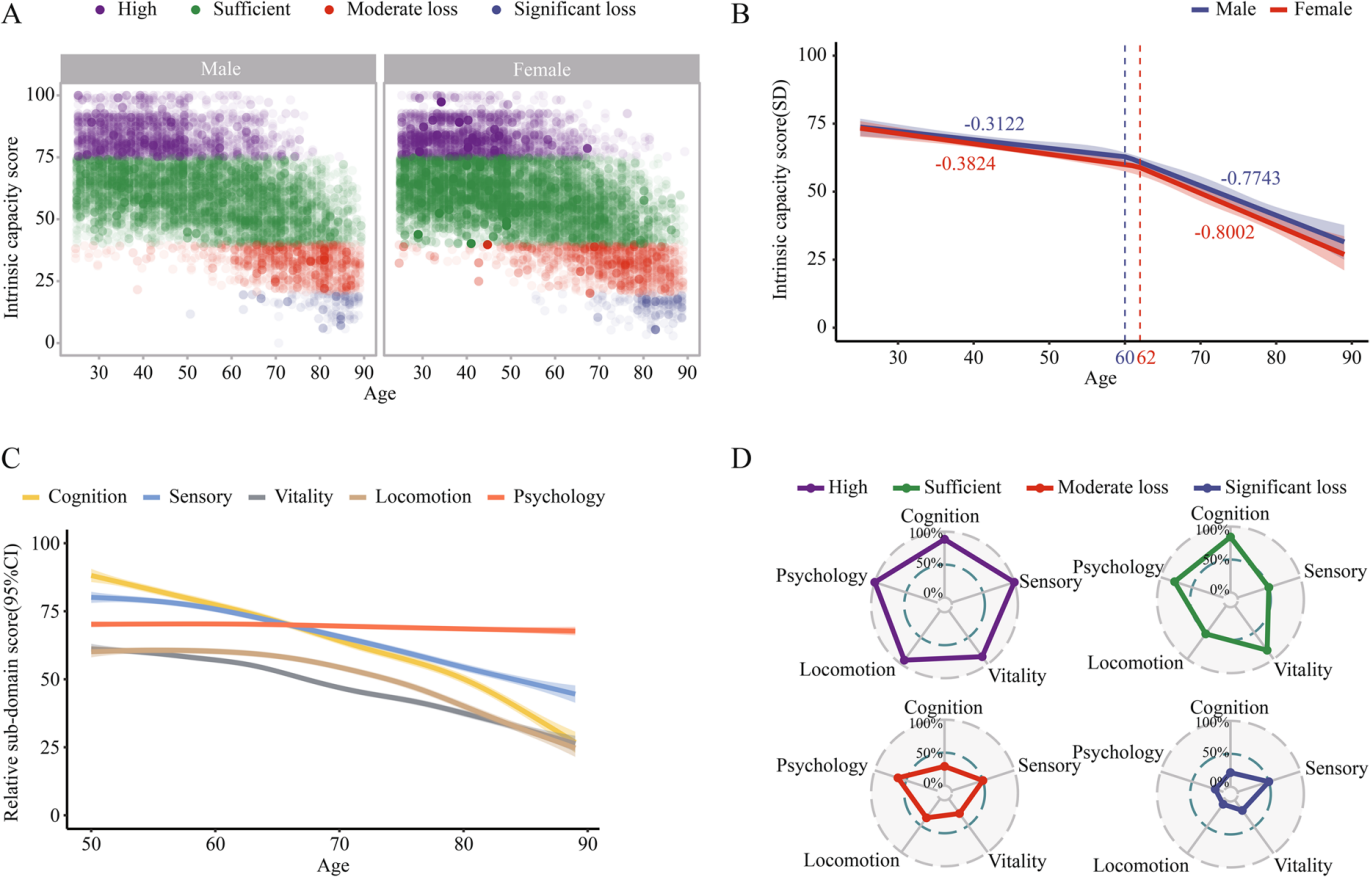
Intrinsic capacity shows a declining trend with age. **A **Age-related distribution of composite IC scores in male and female participants. Higher scores indicate superior intrinsic capacity. Based on the composite IC scores, the participants are stratified into four IC levels (high, sufficient, moderated loss, and significant loss). The purple dots represent participants with high intrinsic capacity (75 ≤ score ≤ 100); green dots represent participants with sufficient intrinsic capacity (40 ≤ score < 75); red dots represent participants with moderate loss of intrinsic capacity (20 ≤ score < 40); blue dots represent participants with significant loss of intrinsic capacity (0 ≤ score < 20). **B** The line chart shows the declining trend and inflection point of IC based on age in both male and female participants. The numbers along the solid lines represent slopes before and after the inflection point in both men and women. The numbers on the *X*-axis at the base of the dotted lines represent the age of male and female participants at the corresponding inflection points. **C** Cross-sectional age trends of the five domains (cognition, sensory, vitality, locomotion, and psychology) in participants aged 50 years and older. **D** Radar chart shows the relative contribution of the five domains towards IC of participants in the four IC levels. The dot represents the percentile of an individual’s score in each domain

The composite IC scores declined steadily with increasing age (Fig. 1A, B, Table S3). A piecewise linear regression model was used to illustrate the age-related trends of composite IC scores (Fig. 1B). At comparable ages, the composite IC scores were slightly higher in men than in women. However, both sexes exhibited a consistent decline in IC with advancing age. Infection points were identified at 60 years for men and 62 years for women (Fig. 1B). After these inflection points, a marked and accelerated decline in IC was observed in both sexes. Statistical tests indicated significant differences in the slopes before and after the inflection points for both men and women (*p* < 0.0001, for both sexes). Although the decline of IC appeared slightly steeper in women than in men, the differences in slopes between sexes were not statistically significant, either before (*p* = 0.044) or after (*p* = 0.356) the inflection point. The average composite IC score was 48.6 ± 17 among participants aged 60 years and above, compared to 67.8 ± 14 among participants aged below 60 years.

Based on the composite IC scores, the participants were further stratified into four IC levels (High, Sufficient, Moderate loss, and Significant loss). The proportion of participants in each IC level is presented in Table 2. In all four IC levels, the age composite differed markedly. Specifically, the proportion of participants with lower IC levels (Moderate and Significant loss) was significantly higher among those aged ≥ 60 years compared to those aged < 60 years (31.6% vs. 2.1%). Conversely, the proportion of participants with acceptable IC levels (High and Sufficient) was significantly lower in the ≥ 60 age group than in the < 60 age group (68.4% vs. 97.9%). Furthermore, in all the four IC levels, a large proportion of older participants under the age of 75 maintained relatively good functional status: 81.84% of participants aged < 75 years were classified as having High or Sufficient IC levels, compared to only 45.12% of those aged ≥ 75 years (Table 2). The proportion of individuals with Moderate or Significant loss of IC increased sharply with advancing age, rising from 40% in the 75–79 age group to 74.3% in the 85–89 age group. Overall, among participants aged ≥ 70 years, the proportion classified as having High IC declined by approximately 50% with every five-year increment in age (Table 2). Age- and sex-specific distributions across the four IC levels are provided in Supplementary Tables S4−1, S4−2, and S4−3. Significant sex differences in the distribution of older participants across ten-year age intervals were also observed (Table S5; *p* < 0.05).

**Table 2:** The proportion of participants categorized by age in different IC levels

Groups	Total	Intrinsic capacity level			
		High	Sufficient	Moderate loss	Significant loss
Age in years					
< 60	10,834	3390/10,834 (31.3%)	7216/10,834 (66.6%)	223/10,834 (2.1%)	5/10,834 (0.0%)
≥ 60	6252	378/6252 (6.0%)	3899/6252 (62.4%)	1677/6252 (26.8%)	298/6252 (4.8%)
50–54	1348	263/1348 (19.5%)	1023/1348 (75.9%)	61/1348 (4.5%)	1/1348 (0.1%)
55–59	1453	254/1453 (17.5%)	1107/1453 (76.2%)	88/1453 (6.1%)	4/1453 (0.3%)
60–64	1414	190/1414 (13.4%)	1081/1414 (76.4%)	139/1414 (9.8%)	4/1414 (0.3%)
65–69	1341	117/1341 (8.7%)	996/1341 (74.3%)	220/1341 (16.4%)	8/1341 (0.6%)
70–74	1210	43/1210 (3.6%)	818/1210 (67.6%)	321/1210 (26.5%)	28/1210 (2.3%)
75–79	978	19/978 (1.9%)	568/978 (58.1%)	360/978 (36.8%)	31/978 (3.2%)
80–84	800	7/800 (0.9%)	307/800 (38.4%)	385/800 (48.1%)	101/800 (12.6%)
85–89	509	2/509 (0.4%)	129/509 (25.3%)	252/509 (49.5%)	126/509 (24.8%)

Data are represented as numbers *n*/*N* (%)

### Domain specific changes and heterogeneity in IC

We further analyzed the age-related decline in the five IC domains: cognition, sensory, vitality, locomotion, and psychology. Based on the restricted cubic spline model analysis, relative scores for four of the five domains (cognition, sensory, vitality, and locomotion) showed a clear decline with increasing age, while the psychological domain remained relatively stable (Fig. 1C). The extent of decline was quantified by calculating the difference in relative scores between the 50–54 and 85–89 age groups for each domain. The degree of decline was greatest in the cognitive domain (60.7%), followed by locomotion (52.9%), vitality (51.0%), and sensory function (39.5%). In contrast, the psychological domain exhibited minimal decline, with a reduction of only 1.7% across the same age range. To visualize the IC profile of individuals with a radar chart (Fig. 1D), linear regression and generalized additive models were used to predict the continuous domain-specific scores (Fig. 1D). This graphical representation facilitates the identification of weaker domains and supports the development of targeted interventions to promote individual IC level.

**Fig. 2:**
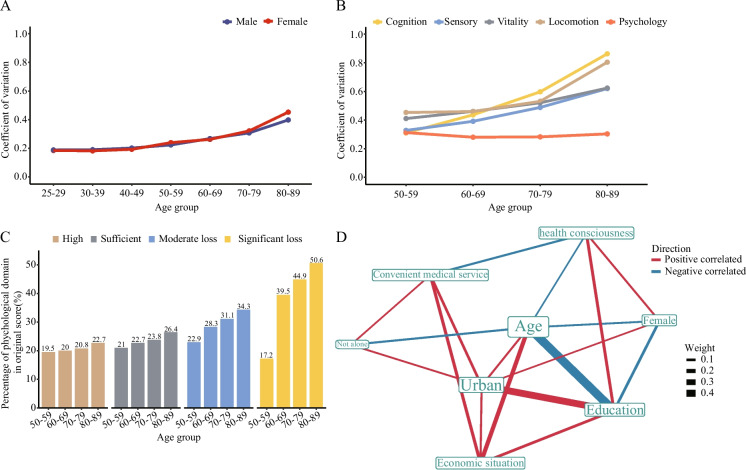
The characteristics and impact factors of intrinsic capacity. **A** The coefficient of variation in the composite IC scores among participants of different age groups. **B** The coefficient of variation for the five IC domain scores in different age groups. **C** The percentages of the psychological domain score in the original IC score among participants of different age groups in the four intrinsic capacity levels. **D** Spearman partial correlation analysis of factors associated with IC. The size of each text label reflects the number of other indicators correlated with the given variable. Red lines indicate positive correlations, while blue lines indicate negative correlations. The thickness of each line is proportional to the absolute value of the correlation coefficient

To assess the heterogeneity of IC, we analyzed the coefficient of variation (CV) of composite IC scores across different age groups. In the three younger age groups (25–29, 30–39, and 40–49 years), the CV values were approximately 0.2. In contrast, among the oldest participants (aged 80–89 years), the CV values increased to around 0.4 (Fig. 2A). The age-related increase in CV indicates greater heterogeneity in IC among older adults, suggesting that the decline in IC with age is not uniform, and that individuals with relatively preserved functional capacity exist across all age groups (Table 2; Table S4−1, S4−2, S4−3).

With the exception of the psychological domain, the CV increased with age in the other four IC domains (cognition, sensory, vitality, and locomotion) (Fig. 2B). The differences in CV values across age groups were statistically significant for the cognitive, sensory, and vitality domains (*p* < 0.0001). For the locomotion domain, no significant difference was observed between the 50–59 and 60–69 age groups (*p* = 0.33), but significant differences emerged in the older age groups (*p* < 0.0001). In the psychological domain, the CV values remained relatively stable across most age groups. A statistically significant difference was observed only between the 70–79 and 80–89 age groups (*p* = 0.016), while no significant difference was found between other adjacent age groups (*p* = 1.0 for 50–59 vs. 60–69 years, *p* = 0.69 for 60–69 vs. 70–70 years).

Although both the relative scores and CV values for the psychological domain remained relatively consistent across age groups, its relative contribution to the original IC scores (the score before the Box-Cox and linear transformations) increased with age across all four IC levels (Fig. 2C). Among individuals in the oldest age group with significant loss of IC, the psychological domain accounted for approximately 50% of the original IC score (Fig. 2C).

### Factors associated with intrinsic capacity

**Fig. 3:**
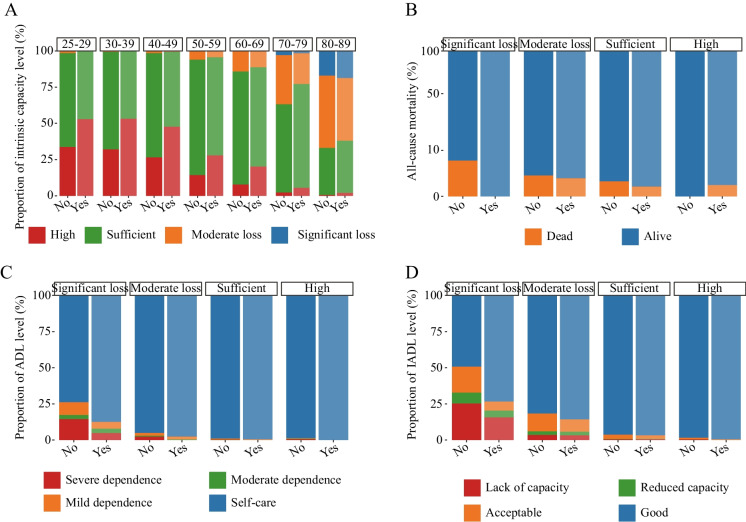
The association of health consciousness on the intrinsic capacity and health outcomes. **A** Proportions of participants across different intrinsic capacity (IC) levels within distinct age groups, stratified by health consciousness status. **B** The crude death rates among participants with or without health consciousness across different IC levels, based on follow-up data. **C** Proportion of participants with varying levels of ADL stratified by health consciousness status across the four IC levels. **D** Proportion of participants with varying levels of IADL stratified by health consciousness status across the four IC levels. “Yes” indicates participants with health consciousness, and “No” indicates those without health consciousness

We conducted a correlation network analysis of factors that significantly associated with IC using Spearman partial correlation coefficients. The results showed that correlations among all factors were generally moderate to high. To account for potential confounding, all factors were included in the subsequent analysis. As all considered factors are categorical, we applied a multivariate ANOVA model, which is equivalent to a linear regression with dummy-coded predictors, and assessed the strength of association using the overall *F*-test *p*-value for each factor. Factors were then ranked based on these *p*-values (Table S6). To ensure the robustness of these rankings, we first examined multicollinearity among the dummy-coded predictors using generalized variance inflation factors (GVIF) adjusted for degrees of freedom and the condition number of the design matrix. Both diagnostics indicated minimal collinearity (all GVIF^(1/(2Df)) ≈ 1; condition number = 5.94). In addition, a group lasso analysis showed that no factor was dropped due to shrinkage. Standardized effect sizes were also calculated to provide a sample-size–independent ranking (Table S7), which was highly consistent with the *p*-value-based ranking. The top three factors most strongly associated with IC scores were age, education level, and health consciousness, with age showing the strongest correlation (Table S6, S7). An ordered linear regression was also conducted to examine linear trends between each factor and the IC score (Table S8). The results were consistent with the ranking based on *p*-values. Furthermore, the factors showed complex interrelationships, as illustrated in the correlation network (Fig. 2D). Associations between factors and IC levels are summarized in Table 3. When comparing individuals across IC levels (from High to Significant loss), lower IC levels were more common among older individuals, females, those living alone, and those with lower socioeconomic status. Conversely, education level and access to convenient health services declined with lower IC levels. Notably, a higher proportion of individuals in the High and Sufficient IC groups reported positive health consciousness (defined as the belief that health is primarily one’s own responsibility) compared to those without such consciousness (Fig. 3A). This finding supports a positive association between health consciousness and IC.

**Table 3:** The characteristics of major factors associated with distinct levels of intrinsic capacity

Variables	Intrinsic capacity levels			
	High	Sufficient	Moderate loss	Significant loss
Age (years)	42.2 [11.8]	52.2 [15.7]	72.9 [11.5]	81.7 [6.8]
Sex ratio^1^	101:100	83:100	80:100	66:100
Residential areas				
Urban	2886/3768 (76.6%)	7747/11,115 (69.7%)	982/1900 (51.7%)	152/303 (50.2%)
Rural	882/3768 (23.4%)	3368/11,115 (30.3%)	918/1900 (48.3%)	151/303 (49.8%)
Education				
Illiteracy	37/3768 (1.0%)	611/11,115 (5.5%)	433/1900 (22.8%)	100/303 (33.0%)
Primary	185/3768 (4.9%)	1378/11,115 (12.4%)	535/1900 (28.2%)	98/303 (32.3%)
Secondary	1643/3768 (43.6%)	5391/11,115 (48.5%)	777/1900 (40.9%)	92/303 (30.4%)
High	1903/3768 (50.5%)	3735/11,115 (33.6%)	155/1900 (8.2%)	13/303 (4.3%)
Living status				
Not living alone	3500/3768 (92.9%)	10,441/11,115 (93.9%)	1619/1900 (85.2%)	237/303 (78.2%)
Living alone	268/3768 (7.1%)	674/11,115 (6.1%)	281/1900 (14.8%)	66/303 (21.8%)
Health consciousness				
No	2204/3768 (58.5%)	8447/11,115 (76.0%)	1550/1900 (81.6%)	239/303 (78.8%)
Yes	1564/3768 (41.5%)	2668/11,115 (24.0%)	350/1900 (18.4%)	64/303 (21.2%)
Economic situation				
Low financial difficulty	806/3678 (21.4%)	2523/11,115 (22.7%)	445/1900 (23.4%)	59/303 (19.5%)
Moderate financial difficulty	2544/3678 (67.5%)	7236/11,115 (65.1%)	1227/1900 (64.6%)	193/303 (63.7%)
High financial difficulty	418/3678 (11.1%)	1356/11,115 (12.2%)	228/1900 (12.1%)	51/303 (16.8%)
Convenient Health service				
Convenient	2944/3678 (80.1%)	8766/11,115 (78.9%)	1527/1900 (80.4%)	206/303 (68%)
Neutral	446/3678 (12.1%)	1428/11,115 (12.8%)	224/1900 (11.8%)	59/303 (19.5%)
Inconvenient	288/3678 (7.8%)	921/11,115 (8.3%)	149/1900 (7.8%)	38/303 (12.5%)

Data are presented as *n*/N (%) or mean [SD]

^1^Females are considered as 100%

Although no significant association was found between health consciousness and all-cause mortality (Fig. 3B), individuals without health consciousness have a high proportion of functional disability in ADL and IADL across all IC levels (Fig. 3C, D). After adjusting for age and sex, health consciousness remained significantly associated with IC status and functional ability: *p* < 0.0001 for IC and ADL, and *p* = 0.004 for IADL.

### Intrinsic capacity and health outcomes

The association between IC levels and health outcomes is shown in Table 4. Higher IC levels were consistently associated with more favorable health profiles. Participants with High and Sufficient IC had lower rates of self-reported disease, disability, and death compared to those with Moderate and Significant loss of IC. Additionally, individuals with High and Sufficient IC levels exhibited more favorable clinical parameters, including higher hemoglobin levels, lower fasting blood glucose, lower systolic blood pressure, and reduced hypersensitive C-reactive protein levels. These associations between IC status and health outcomes remained statistically significant after adjusting for age and sex (Table 4).

**Table 4:** Relationship between IC levels and health outcomes in the included participants

Health outcomes	Intrinsic capacity level				
	High	Sufficient	Moderate loss	Significant loss	*p* value^3^
Rate of self-reported diseases^1^					
None	3036/3768 (80.6%)	7519/11,115 (67.6%)	778/1900 (40.9%)	88/303 (29.0%)	< 0.0001
1 type	495/3768 (13.1)	2388/11,115 (21.5%)	733/1900 (38.6%)	117/303 (38.7%)	< 0.0001
> 1 type	237/3768 (6.3%)	1208/11,115 (10.9%)	389/1900 (20.5%)	98/303 (32.3%)	< 0.0001
ADL^2^					
Independent	894/895 (99.9%)	5986/6029 (99.3%)	1745/1826 (95.6%)	233/303 (76.8%)	< 0.0001
Mild dependent	1/895 (0.1%)	31/6029 (0.5%)	37/1826 (2.0%)	24/303 (7.9%)	< 0.0001
Moderate dependent	NA	6/6029 (0.1%)	13/1826 (0.7%)	9/303 (3.0%)	< 0.0001
Severe dependent	NA	6/6029 (0.1%)	31/1826 (1.7%)	37/303 (12.3%)	< 0.0001
IADL^2^					
Good	882/895 (98.5%)	(5788/6029) 96.0%	(1520/1826) 82.6%	(166/303) 54.8%	< 0.0001
Acceptable	12/895 (1.4%)	(199/6029) 3.3%	(210/1826) 11.5%	(46/303) 15.2%	< 0.0001
Reduced	1/895 (0.1%)	(18/6029) 0.3%	(46/1826) 2.5%	(21/303) 6.9%	< 0.0001
Lack	NA	(24/6029) 0.4%	(62/1826) 3.4%	(70/303) 23.1%	< 0.0001
Clinical parameters					
Hemoglobin (g/L)	144.8 [29.0]	144.0 [20.7]	142.2 [20.4]	136.8 [24.1]	0.143
Fasting blood glucose (mmol/L)	5.2 [1.7]	5.5 [4.8]	5.9 [2.3]	6.0 [2.2]	0.016
Hypersensitive C-reactive protein (mg/L)	1.8 [4.0]	2.4 [11.6]	3.4 [12.0]	5.0 [19.5]	0.0005
Systolic blood pressure (mmHg)	125.2 [17.2]	128.1 [17.5]	139.2 [19.9]	140.0 [23.6]	0.0006
Crude death rate (%)	(1/346) 0.3%	(29/3129) 0.9%	(26/1331) 2%	(10/213) 4.7%	0.0004

Data are represented as *n*/*N* (%) or mean [SD]

^1^Self-reported diseases included hypertension, heart diseases, diabetes, and tumors

^2^ADL and IADL were only examined in people aged 50 years and above

^3^*p* values were calculated between the high plus sufficient groups vs. moderate loss plus significant loss groups and adjusted for age and sex

## Discussion

This study is among the first to characterize age-related changes in intrinsic capacity across adulthood in a Chinese population. Unlike previous studies that mainly focused on older adults [6, 36–44], our study included a broader age range of adult participants, thereby enabling a more comprehensive assessment of IC across adulthood.

In our study, we observed a gradual decline in IC with advancing age, followed by a more pronounced deterioration and greater inter-individual variability after the age of 60 in both men and women. The domains of locomotion, cognition, vitality, and sensory functions exhibited consistent age-related decline, whereas the psychological domain remained relatively stable across the adult life course. In addition to well-established factors associated with lower IC, such as older age, female sex, lower educational attainment, lower income, unmarried status, and urban residence [14, 16, 21–23], we found a positive association between health consciousness and IC, indicating that self-reported attention to health-related behaviors is linked with IC in the studied population.

The concept of IC, proposed by the WHO [3], is a fundamental component of health and healthy aging, emphasizing the preservation of functional ability, which is critical for maintaining well-being in later life. However, healthy aging is not an issue confined to old age. Health status in early and midlife significantly affects health in later life through multiple dimensions, including functional reserve, the accumulation of various health risks, and psychological resilience. Supporting this view, proteomics analyses have shown that age-related biological changes can emerge as early as 34 years of age [45], highlighting the importance of early life interventions. More recently, the WHO has launched a framework to implement a life course approach in practice to healthy aging, which further underscores the necessity of understanding how IC evolved over time. Accordingly, it is of great interest to apply a life course approach to investigate IC, as it enables the identification of key windows for intervention and informs health promotion strategies across different stages of life.

To evaluate IC, we selected domain indicators based on their scientific significance and feasibility in population settings. We employed the entropy weight method (EWM) to generate composite and domain-specific IC scores for participants aged 25–89 years from the PENG ZU cohort. This study is among the first to characterize age-related patterns of intrinsic capacity, as well as domain-specific changes, across the adult life course using a weighted scoring approach as far as we know. Our findings revealed a clear inflection point at approximately 60 years of age, after which the rate of IC decline accelerated. This contrasts with the INSPIRE-T cohort in France, which analyzed cross-sectional baseline data from 975 adults aged 20–102 years in the Toulouse region and reported that IC was markedly lower after age 65 years [25]. In contrast, this study includes 17,086 individuals aged 25–89 years across seven major geographical regions in China, introducing substantial demographic, socioeconomic, and environmental heterogeneity. While the earlier IC transition observed in this study may suggest greater population-level vulnerability, it may also partly reflect methodological and design differences between studies, including recruitment strategy, sample size, and IC measurement protocols. In addition, environmental exposures, access to healthcare, and socio-economic factors could further contribute to these between-population differences.

Higher IC was strongly associated with better health outcomes. Individuals with higher IC levels had significantly lower prevalence of self-reported disease, disability, and mortality. Although our data for self-reported disease and disability are cross-sectional, these findings are consistent with previous longitudinal studies [20, 37, 46–49]. Because self-reported disease status may overlook some affected individuals, we also incorporated objective clinical parameters. Significant associations were observed between lower IC levels and the presence of hypertension and hyperglycemia. Future follow-up survey will further clarify the longitudinal relationships between IC and subsequent health outcomes. In addition to socio-demographic characteristics, we found that health consciousness was positively associated with both higher IC scores and better health outcomes. Participants with strong health consciousness exhibited better functional ability even at similar levels of IC, suggesting a possible buffering effect of health consciousness and behavior. Furthermore, although the psychological domain remained relatively stable across the adult lifespan, it contributed substantially to overall IC, particularly among older adults. This pattern underscores the foundational role of psychological functioning in maintaining IC and aligns with the Socioemotional Selectivity Theory [50], which emphasizes the increasing prioritization of emotional goals in later life. Our results further suggest that emotional well-being and health consciousness are associated with IC maintenance in older adults. While causal relationship cannot be inferred from this cross-sectional study, the observed associations provide a rationale for future research exploring whether interventions aimed at fostering emotional resilience and promoting health consciousness could support IC and functional ability at the population level.

Earlier identification of subtle impairments provides an important opportunity for preventive interventions before irreversible deterioration occurs [2]. We applied a four-level stratification system for IC, which allowed for greater granularity in identifying individuals with early or moderate declines in IC. This stratification was inspired by the framework proposed by the WHO [29], with modifications to improve sensitivity and applicability in our study population, taking into account that functional decline may occur earlier in populations from developing countries compared to those in high-income settings. We classified individuals with IC scores more than 2 standard deviations (SD) below the reference median as experiencing significant loss of capacity and ability. To improve the ability to detect early-stage declines, we extended the threshold for “moderate decline” to a broader range. Correspondingly, we narrowed the range for “sufficient capacity” to a more conservative range. The age-related increase in the CV across the cognitive, sensory, vitality, and locomotion domains highlights the increasing heterogeneity in health trajectories with age [51]. This heterogeneity justified the use of finer stratification to better capture individuals who may be on a declining trajectory despite not meeting conventional impairment thresholds. This refined classification enabled the identification of individuals transitioning from sufficient IC to moderate loss—a critical stage during which timely intervention may be most beneficial. Previously used three-level classification systems [52] lacked the sensitivity to detect this transitional group. Furthermore, our radar chart–based visualization approach provided an intuitive display of domain-specific deficits, thereby supporting more targeted and personalized strategies for intervention.

A key strength of this work lies in the inclusion of a study population with a wide age range, broad geographical coverage, and diverse levels of functioning. In addition, the use of EWM to construct both composite and domain-specific IC scores represents a methodological advantage, which enables a data-driven yet interpretable weighting of IC domains capturing the status of functioning of five domains of IC. This approach provides a more balanced reflection of real-world IC distribution and offers practical utility for clinical and public health settings.

Nonetheless, several limitations should be acknowledged. First, the sample size used to generate IC scores was relatively limited considering China’s large population base. Although the study included participants from seven major geographical regions, providing broad coverage of the Chinese adult population, however, some selection bias cannot be entirely ruled out. This is particularly relevant given China’s wide latitudinal span, substantial altitude variation, and marked regional differences in environmental, demographic, and socioeconomic characteristics, which may influence IC.

Second, while EWM provides a transparent, data-driven weighting scheme, its weights are shaped by variability within the dataset. Thus, higher variability in a domain does not imply greater clinical relevance: for example, the sensory domain showed low variation yet remains important for overall functioning. Moreover, because EWM weights are cohort-specific, the generalizability of this model to other populations remains to be validated. This study demonstrated that data-driven IC scores were significantly associated with disease, ADL, and mortality. Future studies should compare alternative scoring approaches, e.g., equal weighting, factor-analytic approaches, to enhance robustness across populations.

Third, IC assessment in this study is constrained by the measurement of health consciousness. While health consciousness is a complex construct, it was assessed using a single question, which may not fully reflect its contribution to IC. Nevertheless, this study offers valuable guidance for more comprehensive assessments in future research. Moreover, a methodological limitation of the present study pertains to the calculation of IC scores in participants under 50 years of age. Under the established indicator framework, all young participants were assigned to the highest cognitive function tier, a classification that may constrain the variability in the contribution of cognitive function to IC within this age group. Should cognitive assessment tools validated for the full spectrum of adult ages become available, they would capture a broader range of variability in cognitive performance, thereby facilitating a more precise evaluation of how cognitive function modulates IC in this population. Therefore, future research would benefit from adopting cross-age or age-adaptive cognitive domain metrics to better delineate cognitive function’s contribution patterns to IC across the entire lifespan.

Fourth, due to the cross-sectional design of the current study, the IC categories in this study were based on statistically derived cut-offs reflecting the PENG ZU cohort distribution. These categories are sample-dependent and serve as an internal framework to describe IC heterogeneity. We were unable to establish causal relationships between low IC and adverse health outcomes, or between health consciousness and IC. Future longitudinal studies are needed to establish clinically meaningful cut-points linked to functional decline or health outcomes, as well as the influence of health consciousness on IC.

Notably, recent findings from ELSA and CHARLS suggest that newer cohorts enter older age with higher IC levels and experience more compressed declines compared to earlier generations [53]. Longitudinal research is essential to understanding the generational trends and trajectories of IC decline in the Chinese population. Such insights are particularly relevant in the context of China’s rapid socioeconomic, healthcare, and environmental transformations over the past three to four decades, which are likely to shape the patterns and prospects of healthy aging in the coming years.

In summary, this study systematically characterized intrinsic capacity across the adult life course in a regionally diverse and broadly representative Chinese cohort. It provides methodological innovations for IC evaluation and offers important insights into the biological, behavioral, and socio-environmental factors shaping healthy aging. IC declined steadily with age, with an inflection point near 60 years of age, suggesting a critical window for early intervention. The study also highlights the significant contributions of health consciousness and emotional well-being to functional maintenance. Future longitudinal studies are needed to map IC trajectories more precisely and develop evidence-based guidelines and policies to promote IC and healthy aging across diverse populations.

## Supplementary Information

Below is the link to the electronic supplementary material.

ESM 1 (DOCX 636 KB)

## Data Availability

The authors support data sharing, and queries in this regard can be addressed to the corresponding author.
