# Supplementary material for: Intrinsic capacity across the adult lifespan in China: baseline analysis from the nationwide longitudinal PENG ZU cohort
Source: GeroScience. 2026 Feb 25;48(3):4509–27. doi: 10.1007/s11357-026-02111-3 (PMC13356213; doi:10.1007/s11357-026-02111-3)
Supplement: Supplementary file 1 — (DOCX 636 KB) [file 11357_2026_2111_MOESM1_ESM.docx]

**Appendix**

1. **Methods**
   1. **Intrinsic Capacity Assessment**

**1.1.1 Extraction and Recoding of Assessment Indicators**

Conceptually inspired by the framework proposed by WHO^[1]^, an composite intrinsic capacity score was calculated based on the five domains **(**cognition vitality, locomotion, sensory, and psychology**)**.

The selection criteria for the assessed indicators were as follows: (a) The indicators showed strong correlations with other indicators within the corresponding domain of our dataset, as confirmed by correlation analysis; (b) The indicators were easy to measure; and (c) The indicators had been previously reported or validated^[2]^.

Eight indicators belonging to five domains were selected for analysis (Table S1). Each domain consisted of 1-3 indicators.

Cognitive: The Montreal Cognitive Assessment 5-minute protocol (MoCA-5 Mini)^[3]^

Vitality: Body mass index (BMI) and grip strength.

Locomotion: Usual gait speed based on walking for 6 meters.

Sensory: Self-reported hearing impairment with three degrees (no impairment, with impairment but no impact on daily life, with impairment and impact on daily life).

Psychology: DASS21^[4]^, self-rate health satisfaction and self-rate life satisfaction.

MoCA-5 mini was tested in participants aged 50 and above, whereas the remaining seven indicators were measured in all participants.

All the indicators were recoded as 1-4 by their quantiles. Subsequently, the weighted vitality and psychology indicators were aggregated. The recoding criteria did not consider sex and age differences (except for grip strength). The details are shown in Table S1.

**Table S1. The category and recode of intrinsic capacity assessment indicators**

| Recode | domains | | | | | | | | |
| --- | --- | --- | --- | --- | --- | --- | --- | --- | --- |
|  | Cognition | Sensory | Vitality^1^ | | | Locomotion | Psychology^2^ | | |
|  | MoCA | Hearing | BMI  (Kg/m^2^) | Grip Strength  (Kg/mal) | Grip Strength  (Kg/female) | Walking speed  (m/s) | DASS scales^3^ | Self-rated health satisfaction | Self-rated life satisfaction |
| 4 | / | / | Normal (18.5≤x<25) | x≥40 | x≥30 | x≥1.3 | 3 | 9-10 | 9-10 |
| 3 | >=24 | No impairment | Grade 1 overweight (25≤x<27) | 30≤x<40 | 22≤x<30 | 0.9≤x<1.3 | 2 | 6-8 | 6-8 |
| 2 | 15-23 | Impaired but has no impact on daily life | Grade 2 overweight (27≤x<30) | 20≤x<30 | 15≤x<22 | 0.6≤x<0.9 | 1 | 3-5 | 3-5 |
| 1 | <15 | Impaired and has impact on daily life | Underweight and Obesity (x<18.5 & x ≥30) | x<20 | x<15 | x<0.6 | 0 | 1-2 | 1-2 |
| ^1^Vitality = 0.2*BMI + 0.8*Grip Strength  ^2^Psychology= DASS scales + 2.5 * Sefl-rated life satisfaction + 20*Self-rated health satisfaction  Weights in 1 and 2 are determined by using a hybrid approach that combines entropy-based, data-driven weighting with expert-informed adjustments to enhance clinical interpretability.  ^3^DASS scales= D+A+S; D=0: depression score>9, D=1: Depression score≤9; A=0: anxiety score>7, A=1: anxiety score≤7; S=0: stress score>14, S=1: stress score≤14 | | | | | | | | | |

**1.1.2 Determination and Calculation for Entropy Weight**

The Entropy Weight Method (EWM)^[5]^ was selected based on its ability to provide a balanced weighted distribution of the domains, thereby aligning more closely with the actual situation. The formula and results of calculating the weights are shown below:

Step 1: For i-th participant, calculate the proportion of the j-th item among all the items based on the following formula:

$$\rho_{\mathrm{ij}}=\frac{x_{\mathrm{ij}}}{\sum_{i=1}^{n} x_{\mathrm{ij}}};$$

Step 2: Calculate the information entropy and also define $0\ln\left( 0 \right)$=0 as follows:

$$\mathrm{Ent}_{j}=-\frac{\sum_{i=1}^{n} \rho_{\mathrm{ij}}\ln\left( \rho_{\mathrm{ij}} \right)}{ln(n)};$$

Step 3: Calculate the weights as follows:

$$w_{j}=(1-Ent_{j})/\sum_{j=1}^{m} \left( 1-Ent_{j} \right) .$$

The weights calculated for each domain are shown in Table S2.

**Table S2. The weights for each domain in this study**

| Domains | Cognition | Sensory | Vitality | Locomotion | Psychology |
| --- | --- | --- | --- | --- | --- |
| Weight | 0.2026 | 0.1461 | 0.2034 | 0.2417 | 0.2061 |

**1.1.3 Transformation of IC score**

Once the five weights were obtained, the weighted sum of the recoded values for each domain was calculated to derive the original IC score. Then, Box-Cox transformation of the original score was performed to achieve a normal distribution. Subsequently, linear transformation was performed to scale the intrinsic capacity scores to a range of 0-100. Composite IC score was defined as the final score on a scale of 0-100.

Stratified Criteria for IC Levels:

Based on the mean and standard deviation (SD) values of the composite IC scores, the study participants were stratified into four IC levels. The boundaries between neighboring two levels were adjusted for application. The details of classification were as follows:

1. High level: IC score ≥75 (approximately ≥1.5 SD above the mean);
2. Sufficient level: 40 ≤IC score <75 (approximately between −0.75 SD and 1.5 SD of the mean);
3. Moderate loss: 20 ≤IC score <40 (approximately between −2 SD and −0.75 SD of the mean);
4. Significant loss: IC score <20 (approximately <−2 SD of the mean).

**1.1.4 Extrapolation**

MoCA is primarily developed and validated for middle-aged and older adults and is commonly used in individuals aged 50 years and above to detect early cognitive impairment. In our field-based cohort study, the MoCA 5-minute protocol^[3]^ was administered only to participants aged 50 years and older to assess the cognitive domain of IC. To enable the construction of composite IC scores for participants younger than 50, we imputed the cognitive score as the median value of 24 and assigned 3 points, which is supported by the finding that the median value of MoCA score under 60 years was 24^[6]^. We extrapolated the IC assessment to participants below 50 years by applying the same weights as initially calculated for individuals aged 50 and above. This imputation strategy allows for comparisons of IC across the full adult age spectrum while minimizing potential bias due to missing domain-specific data.

**1.1.5 Individual’s Capacity Represented by domain-specific Score (Radar graph)**

To assess the intrinsic capacity of an individual using the radar graph, linear regression and generalized additive models were used to predict the continuous domain-specific scores. Points on the radar graph indicate percentiles and reflect the capacity of the individual in each domain.

1. **Results**

**2.1 Development and validation of composite intrinsic capacity score**

(1) Figure S1 demonstrated the flow chart of composite intrinsic capacity score development process. The distribution and value of IC scores calculated in this study (FigS2) consistent with published report from the WHO^[7]^.


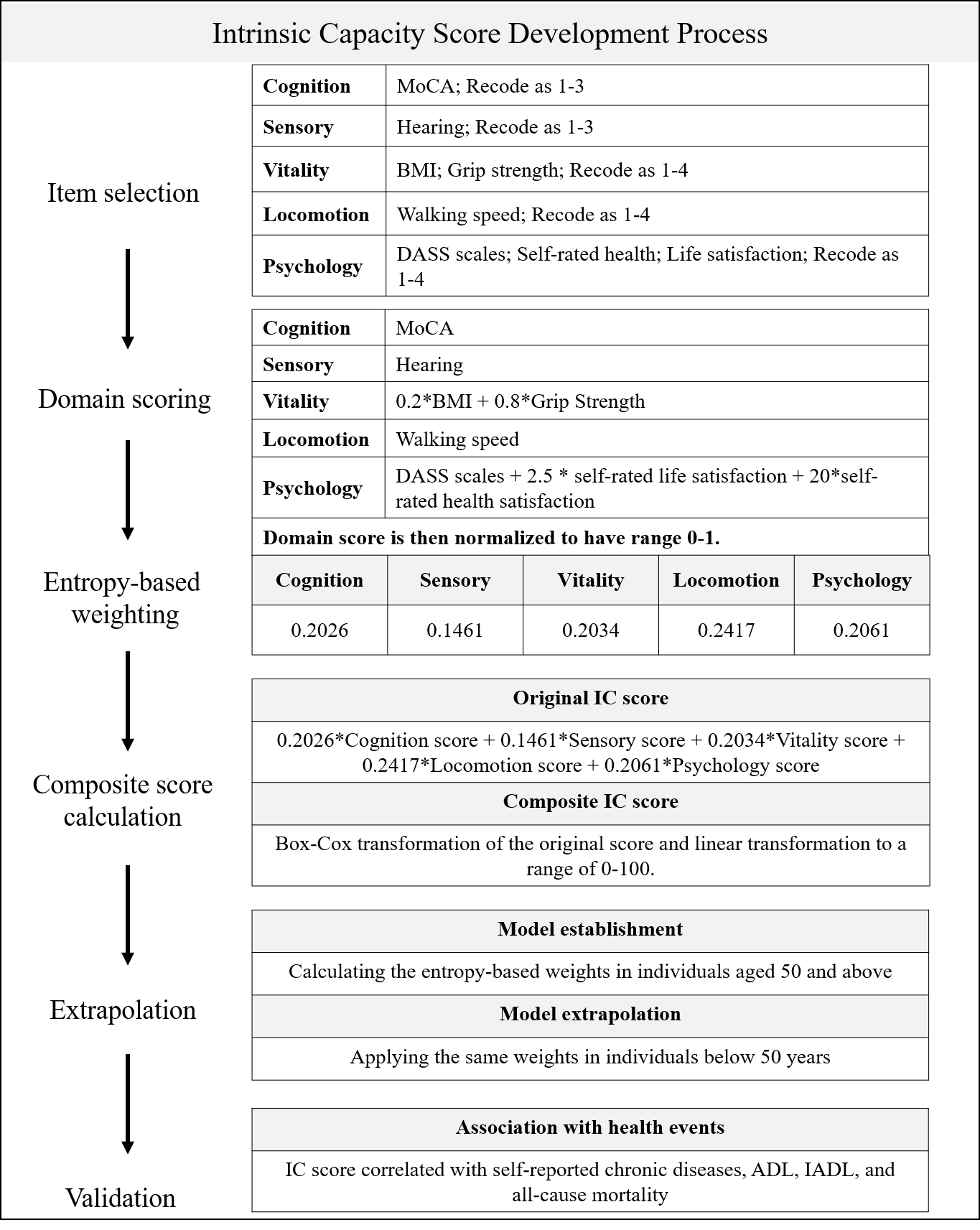


**Figure S1. Flow chart of composite intrinsic capacity score development process.**


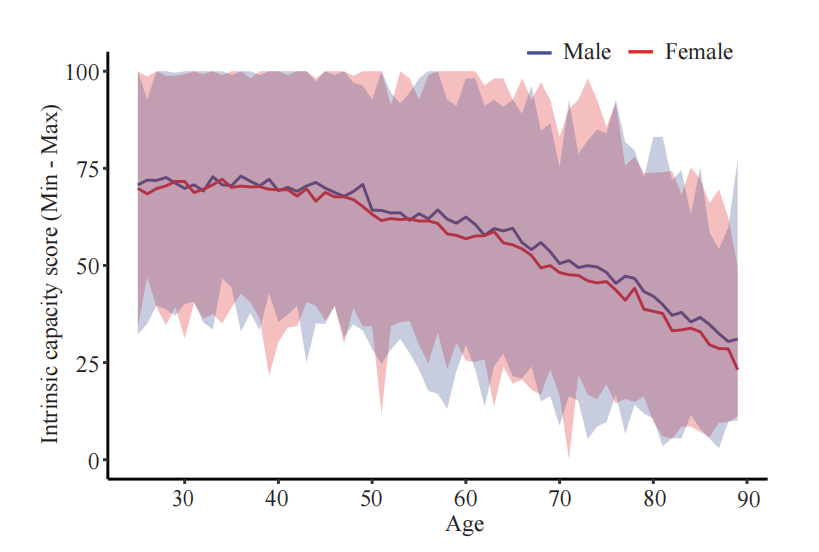


**Figure S2. The distribution and value of IC scores across different ages as calculated in this study.**

(2) We then stratified study participants by the indicator of time to complete five sit-to-stand tests, which was closely related with locomotion but not used for estimating the IC score. The average time to complete five sit-to-stand tests showed significant association with different levels of intrinsic capacity (Fig. S3).


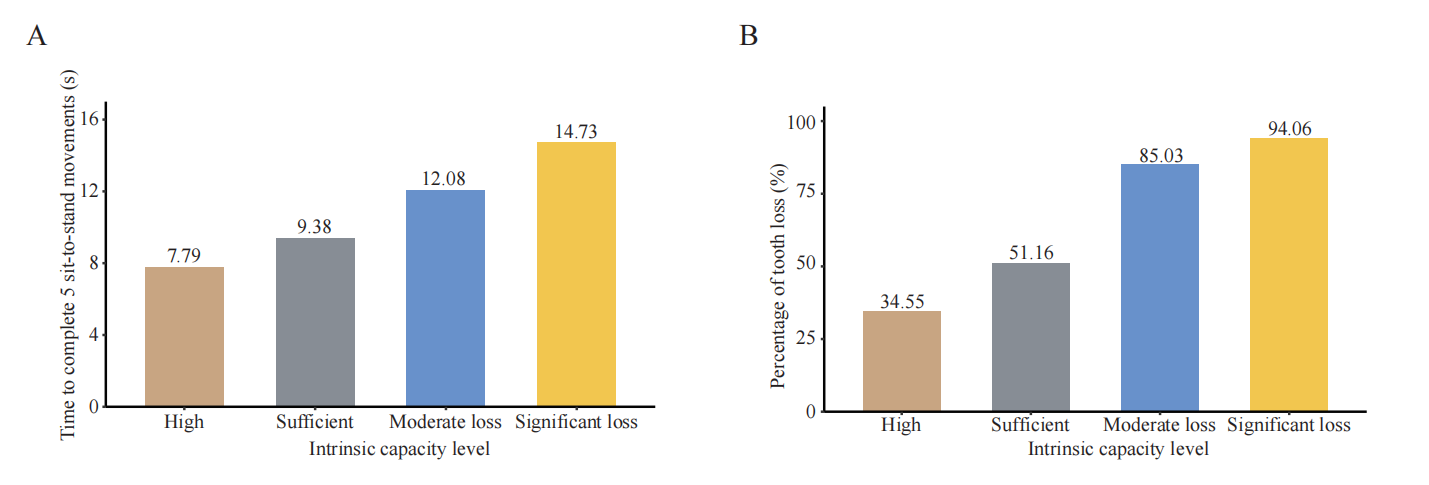


**Figure S3. Validation of intrinsic capacity levels using different indicator.**

**2.3 IC scores by sex and age**

The composite IC scores decreased with age in both males and females (Table S3).

**Table S3. Intrinsic capacity scores stratified by age and sex**

| Groups | Intrinsic capacity score (Mean (SD)) | | |
| --- | --- | --- | --- |
|  | Total | Male | Female |
| Age groups (years) |  |  |  |
| Below 60 years | 67.82(14.1) | 68.7(14.2) | 67.09(13.9) |
| 60 years and above | 48.57(17.0) | 49.98(17.1) | 47.24(16.7) |
|  |  |  |  |
| 25-29 | 70.80(13.2) | 71.64(13.5) | 70.10(12.9) |
| 30-39 | 70.64(13.1) | 70.61(13.7) | 70.67(12.7) |
| 40-49 | 70.78(13.1) | 71.57(13.3) | 70.11(12.9) |
| 50-54 | 69.27(13.5) | 70.04(14.4) | 68.66(12.8) |
| 55-59 | 68.10(13.5) | 69.33(13.7) | 67.08(13.2) |
| 60-64 | 62.67(14.0) | 63.39(13.7) | 62.12(14.2) |
| 65-69 | 61.22(14.8) | 62.65(14.4) | 60.11(14.9) |
| 70-74 | 58.41(14.9) | 59.62(15.4) | 57.33(14.3) |
| 75-79 | 54.16(14.8) | 56.01(15.3) | 52.39(14.0) |
| 80-84 | 48.60(14.6) | 50.24(15.1) | 47.19(14.0) |
| 85-89 | 44.70(14.8) | 46.26(14.3) | 43.17(15.0) |

**2.3 Proportions of IC level by sex and age**

Tables S4-1, S4-2, and S4-3 show the details regarding the proportions of different IC levels among males and females in various age groups. The statistical differences in the proportions of different intrinsic capacity levels based on sex and age are shown in table S5.

**Table S4-1. The proportion of intrinsic capacity levels among males in different age groups**

| Groups (Male) | Intrinsic capacity level | | | | |
| --- | --- | --- | --- | --- | --- |
|  | People | High | Sufficient | Moderate loss | Significant loss |
| Age groups(years) |  |  |  |  |  |
| Below 60 years | 4876 | (1673/4876)34.3% | (3122/4876)64% | (80/4876)1.6% | (1/4876)0% |
| 60 years and above | 3030 | (217/3030)7.2% | (1930/3030)63.7% | (763/3030)25.2% | (120/3030)4% |
| 25-29 | 641 | (268/641)41.8% | (365/641)56.9% | (8/641)1.3% | NA |
| 30-39 | 1517 | (602/1517)39.7% | (904/1517)59.6% | (11/1517)0.7% | NA |
| 40-49 | 1500 | (563/1500)37.5% | (920/1500)61.3% | (17/1500)1.1% | NA |
| 50-54 | 583 | (117/583)20.1% | (448/583)76.8% | (17/583)2.9% | (1/583)0.2% |
| 55-59 | 635 | (123/635)19.4% | (485/635)76.4% | (27/635)4.3% | NA |
| 60-64 | 667 | (103/667)15.4% | (501/667)75.1% | (61/667)9.1% | (2/667)0.3% |
| 65-69 | 656 | (74/656)11.3% | (483/656)73.6% | (95/656)14.5% | (4/656)0.6% |
| 70-74 | 561 | (29/561)5.2% | (395/561)70.4% | (125/561)22.3% | (12/561)2.1% |
| 75-79 | 484 | (10/484)2.1% | (303/484)62.6% | (159/484)32.9% | (12/484)2.5% |
| 80-84 | 394 | (1/394)0.3% | (168/394)42.6% | (188/394)47.7% | (37/394)9.4% |
| 85-89 | 268 | NA | (80/268)29.9% | (135/268)50.4% | (53/268)19.8% |

**Table S4-2. The proportion of intrinsic capacity levels among females in different age groups.**

| Groups (Female) | Intrinsic capacity level | | | | |
| --- | --- | --- | --- | --- | --- |
|  | People | High | Sufficient | Moderate loss | Significant loss |
| Age groups (years) |  |  |  |  |  |
| Below 60 years | 5958 | (1717/5958)28.8% | (4094/5958)68.7% | (143/5958)2.4% | (4/5958)0.1% |
| 60 years and above | 3222 | (161/3222)5% | (1969/3222)61.1% | (914/3222)28.4% | (178/3222)5.5% |
| 25-29 | 775 | (281/775)36.3% | (485/775)62.6% | (9/775)1.2% | NA |
| 30-39 | 1760 | (630/1760)35.8% | (1124/1760)63.9% | (6/1760)0.3% | NA |
| 40-49 | 1840 | (529/1840)28.8% | (1288/1840)70.0% | (23/1840)1.3% | NA |
| 50-54 | 765 | (146/765)19.1% | (575/765)75.2% | (44/765)5.8% | NA |
| 55-59 | 818 | (131/818)16% | (622/818)76% | (61/818)7.5% | (4/818)0.5% |
| 60-64 | 747 | (87/747)11.6% | (580/747)77.6% | (78/747)10.4% | (2/747)0.3% |
| 65-69 | 685 | (43/685)6.3% | (513/685)74.9% | (125/685)18.2% | (4/685)0.6% |
| 70-74 | 649 | (14/649)2.2% | (423/649)65.2% | (196/649)30.2% | (16/649)2.5% |
| 75-79 | 494 | (9/494)1.8% | (265/494)53.6% | (201/494)40.7% | (19/494)3.8% |
| 80-84 | 406 | (6/406)1.5% | (139/406)34.2% | (197/406)48.5% | (64/406)15.8% |
| 85-89 | 241 | (2/241)0.8% | (49/241)20.3% | (117/241)48.5% | (73/241)30.3% |

**Table S4-3. The proportion of intrinsic capacity level in participants of different age group.**

| Groups | Intrinsic capacity level | | | | |
| --- | --- | --- | --- | --- | --- |
|  | People | High | Sufficient | Moderate loss | Significant loss |
| Age groups (years) |  |  |  |  |  |
| 25-29 | 1416 | (549/1416)38.8% | (850/1416)60% | (17/1416)1.2% | NA |
| 30-39 | 3277 | (1232/3277)37.6% | (2028/3277)61.9% | (17/3277)0.5% | NA |
| 40-49 | 3340 | (1092/3340)32.7% | (2208/3340)66.1% | (40/3340)1.2% | NA |
| 50-54 | 1348 | (263/1348)19.5% | (1023/1348)75.9% | (61/1348)4.5% | (1/1348)0.1% |
| 55-59 | 1453 | (254/1453)17.5% | (1107/1453)76.2% | (88/1453)6.1% | (4/1453)0.3% |
| 60-64 | 1414 | (190/1414)13.4% | (1081/1414)76.4% | (139/1414)9.8% | (4/1414)0.3% |
| 65-69 | 1341 | (117/1341)8.7% | (996/1341)74.3% | (220/1341)16.4% | (8/1341)0.6% |
| 70-74 | 1210 | (43/1210)3.6% | (818/1210)67.6% | (321/1210)26.5% | (28/1210)2.3% |
| 75-79 | 978 | (19/978)1.9% | (568/978)58.1% | (360/978)36.8% | (31/978)3.2% |
| 80-84 | 800 | (7/800)0.9% | (307/800)38.4% | (385/800)48.1% | (101/800)12.6% |
| 85-89 | 509 | (2/509)0.4% | (129/509)25.3% | (252/509)49.5% | (126/509)24.8% |

**Table S5. Statistical difference in the proportion of intrinsic capacity levels in males and females of different age groups.**

| Groups | Intrinsic capacity level | | | | |
| --- | --- | --- | --- | --- | --- |
| Age （years） | Gender | High | Sufficient | Moderate loss | Significant loss |
| 60 years and above | Male | (217/3030)7.2% | (1930/3030)63.7% | (763/3030)25.2% | (120/3030)4% |
|  | Female | (161/3222)5% | (1969/3222)61.1% | (914/3222)28.4% | (178/3222)5.5% |
|  | p-value | 0.0014** | 0.034* | 0.0059** | 0.0059** |
| 60-69 | Male | (177/1323)13.4% | (984/1323)74.4% | (156/1323)11.8% | (6/1323)0.5% |
|  | Female | (130/1432)9.1% | (1093/1432)76.3% | (203/1432)14.2% | (6/1432)0.4% |
|  | p-value | <0.0014** | 0.31 | 0.13 | 0.89 |
| 70-79 | Male | (39/1045)3.7% | (698/1045)66.8% | (284/1045)27.2% | (24/1045)2.3% |
|  | Female | (23/1143)2% | (688/1143)60.2% | (397/1143)34.7% | (35/1143)3.1% |
|  | p-value | 0.022* | 0.0026** | 0.0005*** | 0.27 |
| 80-89 | Male | (1/662)0.2% | (248/662)37.5% | (323/662)48.8% | (90/662)13.6% |
|  | Female | (8/647)1.2% | (188/647)29.1% | (314/647)48.5% | (137/647)21.2% |
|  | p-value | 0.024* | 0.0024** | 0.93 | 0.0011** |
| *：0.01 < p-value < 0.05; **: 0.001 < p-value < 0.01; ***: 0 < p-value < 0.001 | | | | | |

p-values for Chi-squared test:

60-years and above: 0.000004***

60-69: 0.002**

70-79: 0.0001**

80-89: 0.00003***

**2.4 Impact factors on intrinsic capacity**

We performed comprehensive analyses of impact factors and their weights. The ranking of the intrinsic capacity impact factors based on p-values is shown in Table S6.

**Table S6 The ranking of impact factors for intrinsic capacity by p-value**

| **Impact Factors** | **p-value** |
| --- | --- |
| Age | <1e-230 |
| Education level | 3.98e-116 |
| Health awareness | 1.94e-43 |
| Self-reporting economic situation | 2.36e-33 |
| Sex | 3.88e-22 |
| Residential aera | 8.90e-06 |
| Utilization of medical service | 2.89e-05 |
| Live alone | 0.038 |

To confirm the ranking based on p-values, we additionally analyzed the standardized effect sizes, which measure the proportion of variance explained by each categorical factor after adjusting for the remaining predictors (Table S7). The resulting ranking of predictors is highly consistent with the ANOVA p-value–based ranking reported in Table S6. The top three factors are the same, and only the 6^th^ and 7^th^ predictors are interchanged, and their effect sizes differ by less than 8 × 10⁻⁵. This consistency further supports the robustness and interpretability of our findings.

**Table S7 The ranking of impact factors for intrinsic capacity by standard effect size.**

| **Impact Factors** | **Standard effect size** |
| --- | --- |
| Age | 0.42 |
| Education level | 0.038 |
| Health awareness | 0.013 |
| Self-reporting economic situation | 0.011 |
| Sex | 6.76e-03 |
| Utilization of medical service | 1.51e-03 |
| Residential aera | 1.43e-03 |
| Live alone | 3.12e-04 |

Furthermore, an ordered linear regression was applied to examine the linear trend of each factor in relation to the IC score (Table S8). The factors were ordered as follows:

- Age: 25–39, 40–59, 60–74, 75–89
- Sex: male < female
- Education: illiterate < primary < secondary < higher education
- Health consciousness: no < yes
- Residential area: rural < urban
- Self-reporting economic situation: difficult < sufficient < affluent
- Living alone: not living alone <living alone
- Utilization of medical service: inconvenient < moderate < convenient

**Table S8 The ranking of impact factors for intrinsic capacity by linear trends of ordered linear regression.**

| **Impact Factors** | **Estimates** | **p-value** |
| --- | --- | --- |
| Age | -23.42264 | <1e-230 |
| Education level | 6.54286 | 1.08e-55 |
| Health consciousness | 2.59688 | 1.12e-44 |
| Self-reporting economic situation | 3.48681 | 1.13e-29 |
| Sex | -1.37875 | 1.03e-15 |
| Residential area | 0.61826 | 0.00165 |
| Live alone | -0.55037 | 0.07951 |
| Utilization of medical service | 0.13189 | 0.6727 |

**References:**

[1] WHO. *Data sources, methods and pooled results for preliminary estimates of intrinsic capacity and functional ability for adults age 60 and over, in 30 countries*. World Health Organization 2020; Available from: <https://cdn.who.int/media/docs/default-source/mca-documents/healthy-ageing/data-methods-and-preliminary-results-for-the-73rd-wha-information-document-2020-04-08.pdf?sfvrsn=da08baf0_2>.

[2] Beard, J.R., et al., *Intrinsic Capacity: Validation of a New WHO Concept for Healthy Aging in a Longitudinal Chinese Study.* J Gerontol A Biol Sci Med Sci, 2022. **77**(1): p. 94-100.

[3] Wong, A., et al., *Montreal Cognitive Assessment 5-minute protocol is a brief, valid, reliable, and feasible cognitive screen for telephone administration.* Stroke, 2015. **46**(4): p. 1059-64.

[4] Lovibond, P.F. and S.H. Lovibond, *The structure of negative emotional states: comparison of the Depression Anxiety Stress Scales (DASS) with the Beck Depression and Anxiety Inventories.* Behav Res Ther, 1995. **33**(3): p. 335-43.

[5] Zhu, Y., D. Tian, and F. Yan, *Effectiveness of Entropy Weight Method in Decision-Making.* Mathematical Problems in Engineering, 2020. **2020**: p. 3564835.

[6] Rossetti, H.C., et al., *Normative data for the Montreal Cognitive Assessment (MoCA) in a population-based sample.* Neurology, 2011. **77**(13): p. 1272-5.

[7] WHO. *World Report on Ageing and Health*. World Health Organization 2015.
